# Novel Triple-Oxygen Isotope Study Indicates Unprecedented Ozone–Particulate Interaction Pathways in Atmospheric Pollution Chemistry

**DOI:** 10.1021/acsomega.4c06957

**Published:** 2025-01-31

**Authors:** Mao-Chang Liang, Chao-Hui Huang, Mark Howard Thiemens, Sourendra Kumar Bhattacharya, Sasadhar Mahata, Yu-Jung Chen, Tai-Sone Yih

**Affiliations:** † Institute of Earth Sciences, Academia Sinica, No. 128, Sec. 2, Academia Road, Nankang, Taipei 11529, Taiwan; ‡ Department of Physics, National Central University, No. 300, Zhongda Rd., Zhongli District, Taoyuan 320317, Taiwan; § Department of Chemistry and Biochemistry, University of California at San Diego, 9500 Gilman Drive,, La Jolla, California 92093, United States; ∥ National Synchrotron Radiation Research Center, Hsin-Ann Road, Hsinchu Science Park, Hsinchu 300092, Taiwan

## Abstract

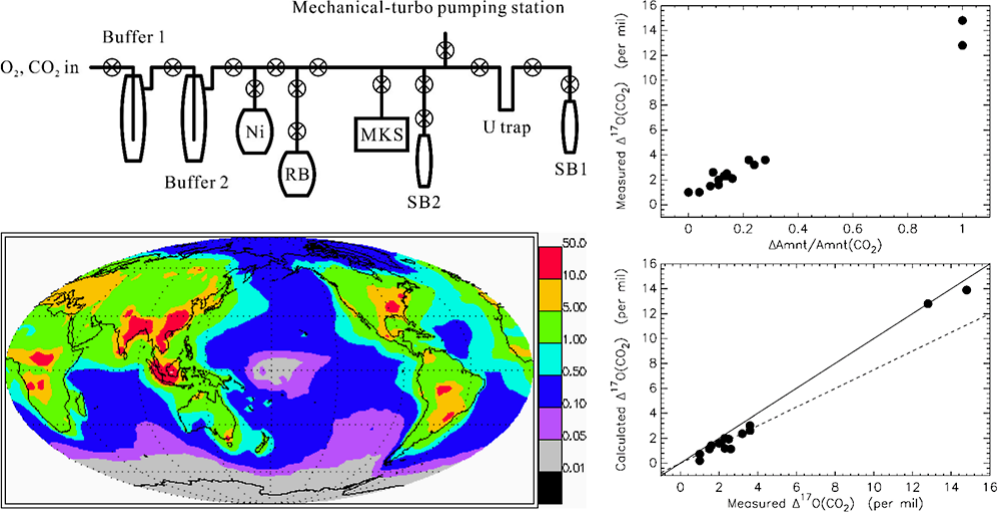

Ozone plays a fundamental role in the chemistry of the atmosphere, mediating oxidation reactions in phases and at phase boundaries. Here, we investigate the least-explored solid-phase heterogeneous processes involving ozone to understand the reaction pathways of O_3_ with airborne aerosols. Using triple oxygen isotope ratios as tracers, we found that the ozone reaction oxidizes organic particles and produces carbon dioxide, with oxygen atoms largely from O_3_. Along with the formation of CO_2_, an equal amount of O_2_ from water decomposition is inferred. Chemical reaction kinetics, however, is yet to be identified. One hypothetical pathway is through Criegee intermediates, formed by the reaction of ozone with aldehyde/ketone-like organic compounds (unsaturated hydrocarbons) catalyzed by metal oxides. Inclusion of the process in a chemistry-transport model could yield a significant change in the ozone budget. The study shows the importance of ozone-induced heterogeneous chemical reactions on aerosol surfaces occurring in polluted atmospheres.

## Introduction

The Earth’s atmospheric environmental system incorporates a complex network of chemical and dynamic processes with interconnected feedbacks.^1^ Despite being a principal component of radiative forcing behind climate change, the direct and indirect effects of aerosols remain the least quantified. They cool Earth’s surface up to 0.5 °C, but uncertainty ranges from 0 to 0.8 °C, compared to an overall warming of 1.1 °C (0.8 to 1.3 °C) observed from 1850 to 1900 to 2010–2019. Moreover, inhaling small-sized particulates smaller than 2.5 μm, commonly referred to as PM_2.5_, can harm human cardiopulmonary and metabolic systems, causing premature deaths.^2^ The climatic and environmental effects and epidemiological impacts of aerosol particles depend on their size and chemical composition.^3,4^ These are difficult to quantify due to their nonuniform emission, inhomogeneous atmospheric distribution, short lifetimes, and notably physiochemical processes that modify the particle reactivity and result in highly complex particulate matter (PM).^5^

Surface heterogeneous chemistry plays a pivotal role in altering the chemical composition of atmospheric aerosols.^6−11^ Aerosols, including particles such as mineral dust, soot, and sea salt, are often coated with a layer of organic compounds and water,^12^ which serve as an interface for chemical reactions. This surface layer absorbs gases, such as ozone, nitrogen oxides, and volatile organic compounds from the surrounding atmosphere.^13,14^ Close proximity on the surface of the aerosol particles of water vapor and the atmosphere promotes heterogeneous reactions leading to formation of new chemical species, such as nitrates, sulfates, and organic aerosols.^5,15^ Contrary to previous studies that showed heterogeneous reactions of ozone with sea salt-dominated or synthetic “clean” particles to be unimportant,^11,14,16^ this work demonstrates that heterogeneous ozonolysis of PM from “polluted” atmospheres is significant. Such an oxidation process has a considerable impact on models of atmospheric chemistry and on the development of air quality policies.

In their experiments dealing with products at the surface of a metal oxide particle, Baltrusaitis and Grassian^6^ showed the reactivity of “wet” interfaces in mediating and boosting chemical reactions. Shaheen et al.^4^ showed that the oxygen isotope anomaly (termed as Δ^17^O, defined as δ^17^O – 0.52 × δ^18^O) of O_3_ is transferred to newly reformed carbonates on the surface of particles in the presence of water and proposed this as a viable process responsible for the observed oxygen isotope anomaly in atmospheric and Martian carbonates.^4,17^ Two mechanisms were hypothesized to sequester the carbonate anomaly from ozone: (1) direct isotope exchange between pre-existing nanocarbonate grains on the surface and O_3_ and (2) formation of new carbonate phases on the grain surface in the presence of CO_2_. Both processes require surface water, and the establishment of a liquid layer enhances chemical reaction rates. In addition, it is known that oxygen-bearing mineral aerosols (such as CaO, Fe_2_O_3_, ZnO, and Cu_2_O), naturally derived from dust, sea spray, and anthropogenic emission, enhance chemical oxidation.^6,18^

Here, we conduct new laboratory experiments to investigate O_3_ oxidation processes occurring in airborne PM containing organics and metal oxides and their kinetics to assess their feedback to the budget of tropospheric O_3_ and its impact on the oxidation state of PM.

## Methods and Experimental Setup

We collected PM for 1 day in the wintertime by pumping air through a Whatman grade 41 cellulose-fiber filter (8″ × 10″) using a Tisch high-volume TSP aerosol sampler in the main campus of Academia Sinica in Taipei, Taiwan. The PM retained on the filter was used as a reagent for chemical reactions with ozone in the laboratory. Reactions of the filter-trapped particles with ozone were allowed to proceed in the dark in a glass chamber of the vacuum system by introducing O_3_ aliquots prepared just prior to the reaction. The triple oxygen isotope composition of the ozone was predetermined and served as a reaction tracer. The two main gaseous products, namely, O_2_ and CO_2_, were collected (along with residual O_3_ in two cases) and analyzed for their isotopic compositions. See the Supporting Information for experimental and analytical details.

## Results and Discussion

**Figure 1:**
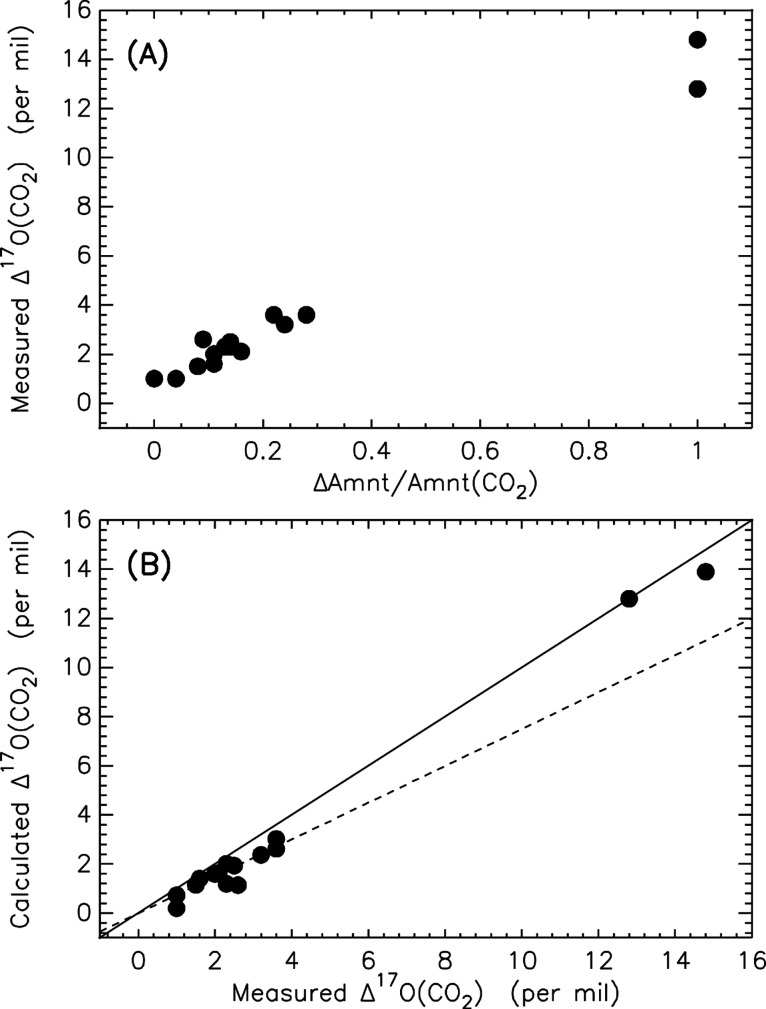
(A) The Δ^17^O values of CO_2_ are a function of CO_2_ produced against the ratio of the excess and total amount of CO_2_ (ΔAmnt/Amnt(CO_2_)). The two are well correlated with *R*^2^ = 0.98. The more the CO_2_ produced, the higher the Δ^17^O values. The experimental points fall along a line, showing that the Δ^17^O value of the final CO_2_ is determined by two end members: excess CO_2_ with nonzero Δ^17^O produced from O_3_ oxidation and the pre-existing CO_2_ with essentially zero Δ^17^O. (B) Following the binary mixing calculation of the aforementioned two endmembers, the experimental data are well reproduced, following closely the slope of unity (solid line). The least-squared linear regression yields a slope of 0.99 ± 0.03 and an intercept of 0.66 ± 0.13 with *R*^2^ = 0.99. The slope of 3/4 denoted by the dashed line is expected if the terminal ozone atoms are only involved.

**Figure 2:**
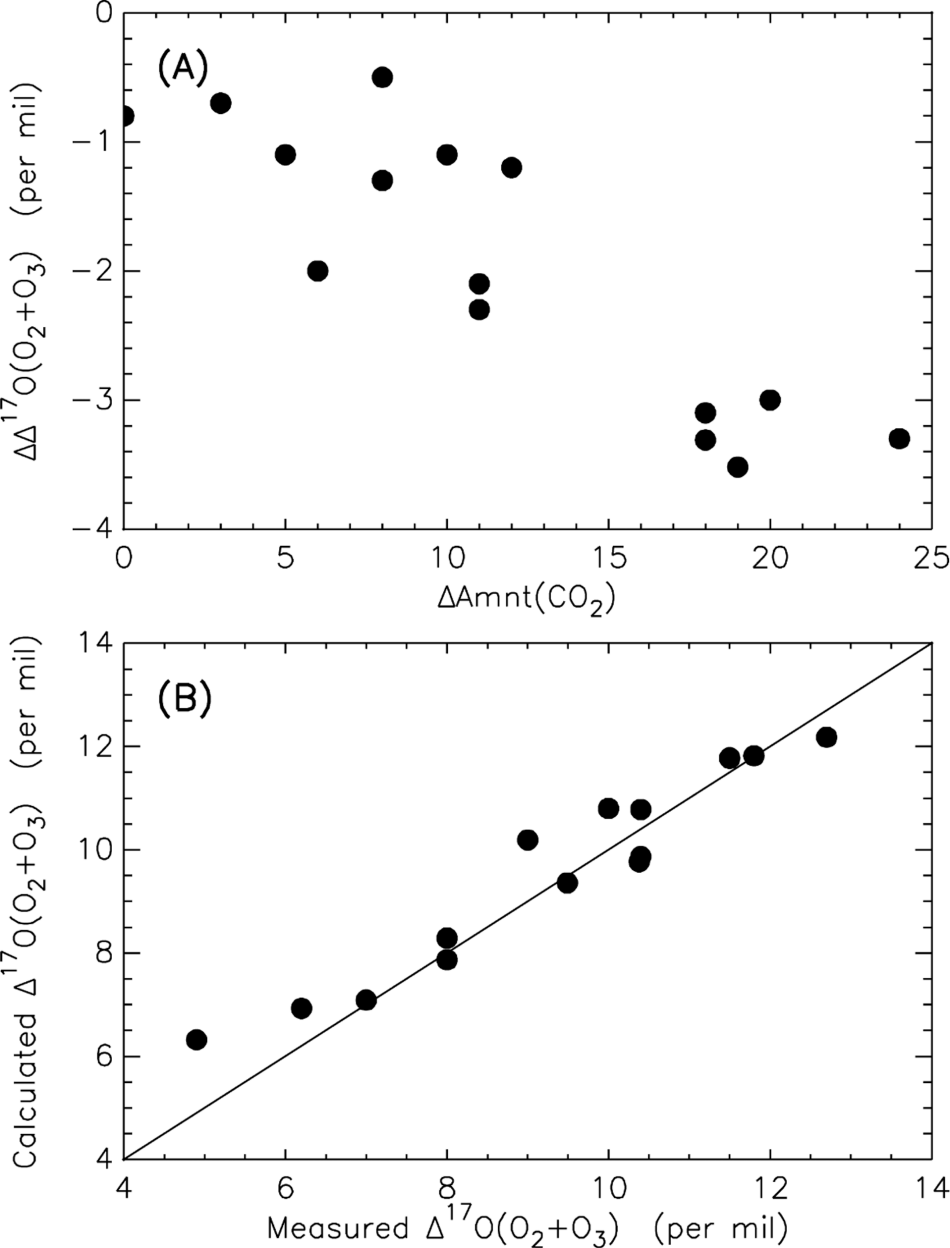
(A) The deviation of the Δ^17^O value of the final O_2_ + O_3_ from the initial O_3_ (ΔΔ^17^Ο(Ο_2_ + Ο_3_)), as a function of the amount of CO_2_ produced (ΔAmnt(CO_2_)). The *R*^2^ value (with intercept zero) is 0.94. This suggests that in the final O_2_ + O_3_, there are two components: ozone-derived (counting O_2_ + O_3_) one having high Δ^17^O from the initial O_3_ and another one with an amount likely affected by the new CO_2_ with a different Δ^17^O value. (B) Assuming simple two-component mixing (from O_3_ with nonzero Δ^17^O and from water with zero Δ^17^O; see text for details), the experimental values of the final O_2_ + O_3_ are well reproduced, following closely the solid line (1:1). The least-squared linear regression (with zero intercept) yields a slope of 0.98 ± 0.02 with *R*^2^ = 0.99.

We observe the following interesting results. First, we found that the ozone fraction remaining after 48 h of the reaction is ∼17% (experiments 38 and 39 in Table 1). This contrasts with a ∼10 h decomposition time of ozone in a PM-free reaction vessel (see Supporting Information Figure S4 and Huang et al.^19^ for the preparation and characterization of ozone). This implies that ozone can be efficiently retained on wet particle surfaces for long periods, mediating and enhancing oxidation of organic compounds.^5,20^ Second, a significant part of the O_3_ oxidizes PM to produce CO_2_ and O_2_, and this new CO_2_ largely carries the Δ^17^O value of the initial O_3_ (Figure 1), likely affected by the concentration of initial CO_2_, if present. There is no evidence of other oxidized gaseous compounds present (e.g., no CO formed) nor incorporation of oxygen into the PM. Third, the product of O_2_ is of a similar amount as the new CO_2_, and this O_2_ possesses a Δ^17^O value of zero (Figure 2). We believe that this O_2_ is derived from the water present at the surfaces of the particles. Details follow.

**Table 1:** Summary of the Results with Isotope Ratios and the Concentrations^a^

expt. no.	initial O_3_			final O_3_ + O_2_			initial CO_2_	new CO_2_	final CO_2_			
	Amnt.	δ^18^O	Δ^17^O	Amnt.	δ^18^O	Δ^17^O	Amnt.	Amnt.	Amnt.	δ^13^C	δ^18^O	Δ^17^O
Ozone with CO_2_ and PM												
17	95	35.0	10.8	84	33.7	10.0	65	0	65	–32.5	36.5	1.0
18	92	39.8	12.2	85	38.9	11.5	65	3	68	–32.6	36.5	1.0
22	91	49.0	11.0	81	49.8	9.0	63	6	69	–32.5	37.0	2.6
23	94	52.1	12.5	80	55.0	10.4	67	11	78	–32.1	37.5	2.5
24	94	53.8	13.1	82	58.8	11.8	66	8	74	–32.3	37.0	2.0
25	94	57.1	13.8	85	59.6	12.7	65	10	75	–32.2	37.5	2.3
29	90	40.3	12.6	77	39.3	11.5	60	5	65	–32.5	36.3	1.5
30	94	44.2	9.2	83	44.1	8.0	63	12	75	–32.3	37.3	2.1
31	91	48.4	10.9	85	47.6	10.4	63	8	71	–32.9	36.7	1.6
32	119	41.2	7.2	90	40.0	4.9	67	11	78	–32.6	36.4	2.3
34	90	48.5	11.1	71	52.7	8.0	62	18	81	–32.3	35.2	3.6
36	99	43.7	9.2	81	48.3	6.2	63	20	83	–31.6	35.0	3.2
37	102	47.0	10.3	77	52.4	7.0	62	24	86	–32.1	34.2	3.6
Ozone with PM only												
38	87	52.7	12.8	15(O_3_)	85.9	12.9	0	18	18	–27.4	35.3	12.8
				***52(O***_***2***_***)***	***48.2***	***8.5***						
39	83	56.7	13.9	13(O_3_)	98.1	15.3	0	19	18	–26.7	34.7	14.8
				***51(O***_***2***_***)***	***53.3***	***8.8***						

a See the Supporting Information for all the data. Amounts (Amnt.) show the oxygen in the species of interest in μmole O_2_ equivalent. All the oxygen isotope values, referenced to VSMOW and δ^13^C to PDB, are in ‰. Δ^17^O is defined by δ^17^O – 0.52 × δ^18^O. Note: In expt. nos. 22 to 37 remaining O_3_ and product O_2_ were clumped together, whereas in nos. 38 and 39, they were separately measured.

### Hypothetical Mechanistic Pathways

The reactions proceed by following the steps outlined below (R1 to R4), with surface water decomposition (R2) intimately linked to the O_3_ heterogeneous reaction (R1). The symbols are H_2_O_s_, surface water on PM, H_2_O_g_, mobile water, likely in the vapor phase, M, a third body including wall surface to quench/decompose O_3_, O′, indicative of the oxygen that has a Δ^17^O value different from that of O_3_. R1 and R2 are net reactions occurring on the surface, likely catalyzed by metal oxides, and R3 and R4 can be gaseous or heterogeneous phases.
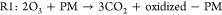

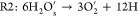

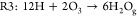

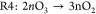

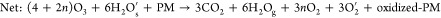


That is, two ozone molecules with 6 surface water molecules reacting with organic compounds produce 3 CO_2_ and 3 O_2_ molecules, with CO_2_ from O_3_ and O_2_ from H_2_O possibly through multiple pathways and not in a single concerted reaction. We observe that the CO_2_ has the same Δ^17^O value as the O_3_, and O_2_ has the value of zero. However, if CO_2_ is present initially, the final CO_2_ has a value 3/4 that of O_3_, but the O_2_ is the same as the CO_2_-free condition.

Table 1 summarizes the experimental results (see Table S2 for the full data). In several experiments, we introduced CO_2_ (∼60 μmol) along with O_3_ to the PM-containing reaction vessel (Table 1). The final CO_2_ (initial plus the reaction produced) Δ^17^O value varies from 1.6 to 14.8 (Table 1) and can be well explained by the presence of two end members: excess CO_2_ with nonzero Δ^17^O produced from O_3_ oxidation in which bulk O_3_ is fully or partially involved and the introduced pre-existing CO_2_ with essentially zero Δ^17^O (Figure 1). That is, the new CO_2_ is solely obtained from the oxidation of O_3_ with organics. This two-component mixing model is further validated by changes in the δ^13^C value of CO_2_ (see the Supporting Information). We did not find the signature of any significant oxygen isotope exchange occurring (between CO_2_, O_3_, and surface water) at the surface of PM, in contrast to the previous findings.^4,6^ A likely explanation is that in our case, the reaction of the O_3_ with “polluted” hydrated PM has a higher reaction rate than any exchange reactions with carbonates and metal oxides. This is also a possible reason that previous studies^11,14,16^ showed low O_3_ reaction probability with water-free clean particles. This study demonstrates the high reactivity of O_3_ with wet polluted PM and suggests the importance of the ozone–particulate reaction process in controlling the budget of O_3_ and the significant role of ozone oxidation in enhancing the oxygen-to-carbon ratios in organic aerosols in the polluted atmosphere.^5,20^

In general, the oxygen atom amount (sum of O_2_, O_3_, and CO_2_) is materially balanced between the before and after reaction states but apparently, the overall Δ^17^O value is not. Analysis of isotope mass balance shows that the apparent imbalance is from O_2_, a product of oxidation and O_3_ decomposition. We can see clearly from Table 1 that along with CO_2_, there is significant production of O_2_ that has a different Δ^17^O value from that of O_3_. Figure 2A shows that greater CO_2_ production (indicated by the Δamnt of CO_2_) is associated with a lower ΔΔ^17^O value of the total O_2_ (deviation from the value of the initial O_3_) where the lowering is due to more of the new O_2_ (reaction-produced O_2_ relative to that from O_3_ decomposition). This connection between CO_2_ and O_2_ is intriguing. The O_2_ results can be well explained by two endmembers contributing to the production of O_2_: one from pure O_3_ dissociation (R4) with the same Δ^17^O value as the bulk O_3_ and one from surface water (called new O_2_) possessing a Δ^17^O value of zero (R2). Taking the fraction of the new O_2_ the same as the CO_2_ amount produced reproduces the observed reduction in O_2_ Δ^17^O (Figure 2B). The correlation is apparent in experiments where the remaining O_3_ and the reaction-produced O_2_ are separated (see Table 1, experiments 38 and 39). We find that the depletion occurs in O_2_ but not in O_3_. In these two cases, the remaining O_3_ has the same Δ^17^O value as the initial O_3_, suggesting that the loss of O_3_ does not introduce any change in the remaining O_3_; it is simply mass-dependent following the slope of 0.52 in the dual oxygen isotope plot (δ^17^O vs δ^18^O). This suggests strongly that all three atoms in O_3_ participate equally in the oxidation processes (i.e., production of CO_2_ and likely mobile water; see more below).

The CO_2_ isotope values are inconsistent with the expected values based on general hypotheses in gas phase chemistry where terminal oxygen atoms in O_3_ possess a higher reaction probability than the central. Moreover, the concurrent production of O_2_ and CO_2_ suggests that the two products are intimately connected. One hypothetical scheme could be through a Criegee intermediate (CI) as follows, providing a source of reactive oxygen with Δ^17^O equal to zero (from the central oxygen atom in O_3_) (R5). First, CI is formed in the ozonolysis of alkenes^21^ (R6). At the surface of particles, likely in the presence of metal oxides, an aldehyde or a ketone (R_2_CO) may be decomposed to a hydrocarbon and CO, similar to that found at high temperatures^22^ (R7). A CI could then catalytically oxidize CO to CO_2_ and produce an aldehyde or a ketone (R_1_CO),^23^ which may then further decompose to R_1_ and CO, following R6.
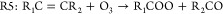

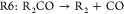

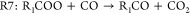

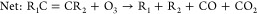
where R_1_ and R_2_ denote arbitrary functional groups. The net result is equal production of CO and CO_2_. The CO from the reactions will have a Δ^17^O 3/2 that of O_3_, and a CO_2_ 3/4. The mechanism explains the data for cases where CO_2_ is present initially (see Figure 1, dashed line) but not the ones without prior CO_2_ which requires all oxygen atoms in O_3_ to participate equally in the production of CO_2_ (Figure 1, solid line). In view of this issue, we hypothesize that O_2_ is derived via the following reactions
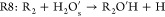

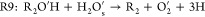

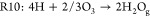

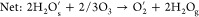


Overall, the net result is that ozonolysis of one alkene will produce 1.5 CO_2_, 1.5 O_2_ (through the conversion of 3H_2_O’ molecules, R2), and three mobile H_2_O molecules (which inherits the Δ^17^O value from O_3_). It is possible that hydrogen peroxide^4^ is an intermediate of the reactions (R9 and R10) in producing O′_2_ and H_2_O_g_ where the presence of H catalyzes the decomposition of O_3_, providing a source of O_2_. We believe all of the above reactions occur and are confined in the surface layer because other gaseous molecules such as CO and H_2_ were not detected after reactions.

In short, O_3_ + PM reactions produce CO_2_ with the value of Δ^17^O largely related to that of the reactant O_3_ (Table 1 and Figure 1), and the production is altered by the prior presence of CO_2_. Surprisingly, the CO_2_ signal observed here is similar to that for CO produced in gas phase ozonolysis,^24^ where CO has a Δ^17^O value close to the bulk O_3_; the main difference is that CO is produced in abundance in the previous experiments and not CO_2_. The branching ratio of the CO_2_ production channel varies between 3 and 24% (on average 12%) of the initial O_3_ based on the amounts measured (Table 1). This suggests that the reaction probability to form CO_2_ is significant, compared to the decomposition branch of O_3_. Along with CO_2_, production of an equal amount of O_2_ with a Δ^17^O value of nearly zero is inferred (Figure 2) indicating the origin from water.

### Atmospheric Implications

**Figure 3:**
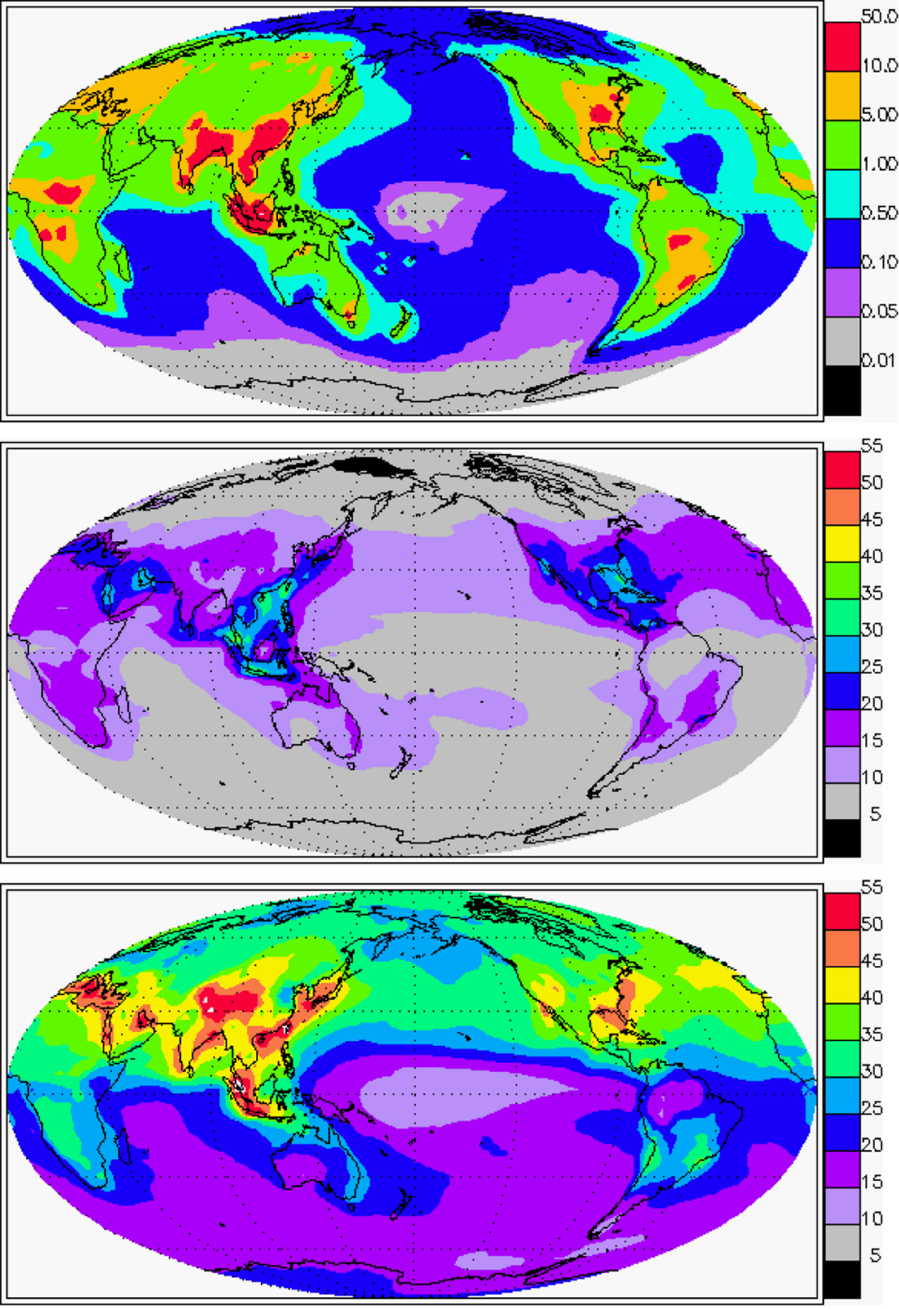
MOZART-4 simulation results with the inclusion of O_3_ + PM at the reaction probability γ_O3_ 0.001; see the Supporting Information for the results with reduced γ_O3_. **(Top)** The annually averaged heterogeneous reaction rate (μgC/m^3^/day) that oxidizes PM to CO_2_, compared to a typical ∼10–100 μg/m^3^ PM loading in the polluted atmosphere. **(Middle)** The annually averaged surface O_3_ concentration (ppb). **(Bottom)** The annually averaged surface O_3_ concentration (ppb) from the standard model. (The figures are made using the GEOV geophysical visualization tool. Copyright: University Corporation for Atmospheric Research and Max Planck Institute for Meteorology, Hamburg.)

To assess the significance of the heterogeneous reactions in the ozone budget and chemical modifications in PM in the atmosphere, we include the reactions in a chemistry-transport model, MOZART-4 (Model for Ozone and Related Chemical Tracers, version 4^9^), driven by the National Centers for Environmental Prediction analysis meteorology (see the Supporting Information for details). From the literature,^25^ the reaction probability could be as high as 10^–2^. As a trial, we take a value of 10^–3^ and run the model; the reduced value at 10^–4^ is shown in Figure S5. We found that the reactions could change the surface ozone concentration significantly (as much as 50%) and cause a significant loss of carbon in PM (Figure 3). Assuming the reaction is confined in the planetary boundary of 100 m, the process will result in 32 TgC/year loss of carbon in the PM globally (Figure 3). For comparison, the organic aerosol production in MOZART-4 is ∼50 Tg/yr^9^ and more (∼100 Tg/yr or more) in new models.^10^ The possibility of having significant O_3_ loss via PM was suggested previously,^8^ but later studies suggested it to be low.^11,14,16^ Our data, in contrast, show that the O_3_–PM reaction probability could be quantitatively significant.

In summary, ozone surface heterogeneous chemistry explored for the first time using the unique isotope anomaly of the ozone quantitatively identifies reactions that play an important role in atmospheric chemistry. High ozone reaction probability is found for PM from polluted atmospheres, in contrast to previous studies made on sea salt-dominated aerosols and synthetic particles that show the reaction is insignificant. In short, we found that the oxygen concentration (sum of O_3_, CO_2_, and O_2_) is balanced with no need to consider water, but the oxygen isotope anomaly (Δ^17^O) is not conserved. The imbalance of Δ^17^O can be fully explained by having water included. The ozone oxidation produced CO_2_, with oxygen atoms in CO_2_ being solely from O_3_. Along with CO_2_, the same amount of O_2_ as CO_2_ was inferred but with oxygen from water. More interestingly, we noticed that ozone in the presence of PM “survives” longer, extending and enhancing the role of ozone in atmospheric chemistry and concomitant adverse health-associated oxidative effects. Furthermore, rather than a general mechanistic scenario of oxidation where oxygen is incorporated into PM, carbon loss is instead observed to be significant in enhancing the O/C ratio of PM. The impact of the new chemistry on the tropospheric ozone budget and organic aerosols’ O/C ratios is demonstrated in a chemistry-transport model. This study suggests that further exploration using this new isotope methodology is essential to accurately determine the ozone–particulate reaction probability.

## Supporting Information Available

Additional experimental details, materials, methods, results, and further discussion (PDF)
