# Supplementary material for: Novel Triple-Oxygen Isotope Study Indicates Unprecedented Ozone–Particulate Interaction Pathways in Atmospheric Pollution Chemistry
Source: ACS Omega. 2025 Jan 31;10(5):4388–94. doi: 10.1021/acsomega.4c06957 (PMC11822502; doi:10.1021/acsomega.4c06957)

Supporting Information

Novel triple-oxygen isotope study indicates unprecedented ozone-particulate interaction pathways in atmospheric pollution chemistry

Mao-Chang Liang[1*], Chao-Hui Huang[2#], Mark Howard Thiemens[3], Sourendra Kumar Bhattacharya[1], Sasadhar Mahata[1], Yu-Jung Chen[2], Tai-Sone Yih[2]

[1]Institute of Earth Sciences, Academia Sinica, No. 128, Sec. 2, Academia Road, Nankang, Taipei 11529, Taiwan

[2]Department of Physics, National Central University, No. 300, Zhongda Rd., Zhongli District, Taoyuan 320317, Taiwan

[3]Department of Chemistry and Biochemistry, University of California at San Diego, 9500 Gilman Drive, La Jolla, CA 92093, USA

[#]Now at National Synchrotron Radiation Research Center, Hsin-Ann Road, Hsinchu Science Park, Hsinchu 300092, Taiwan

*Correspondence to: mcl@gate.sinica.edu.tw

Experimental details and results

We express the oxygen isotope ratios of samples (R_{sample}) in the usual delta notation (in ‰ relative to VSMOW) defined as $\delta^xO=[R_{sample}/R_{VSMOW}-1]$ and isotope ^{17}O-excess ($\Delta^{17}O$) as deviation of the sample composition from the terrestrial mass dependent line,

$$\Delta^{17}O=\delta^{17}O-0.52\times\delta^{18}O. \quad (S1)$$

Mass-balance calculation

We show in the main text that the variation in the isotope composition of two major products, O_2 and CO_2, can be well represented by mixing of two endmembers as described by the following

equations. Instead of the simple $\delta^{17}O$ and $\delta^{18}O$ which are influenced by isotope fractionation caused by chemical reactions and uncertainty in surface water isotope composition, we use the ^{17}O-excess parameter, $\Delta^{17}O$ (defined above), which remains invariant in chemical reactions.

$$\Delta^{17}O(CO_2) = \frac{\Delta_1^{17}O(CO_2)\times[CO_2]_1+\Delta_2^{17}O(CO_2)\times[CO_2]_2}{[CO_2]_1+[CO_2]_2} \qquad \text{(S2)}$$

$$\Delta^{17}O(O_2) = \frac{\Delta_1^{17}O(O_2)\times[O_2]_1+\Delta_1^{17}O(O_3)\times[O_3]_1+\Delta_2^{17}O(O_2)\times[O_2]_2}{[O_2]_1+[O_2]_2} \qquad \text{(S3)}$$

In addition, we examine changes of $\delta^{13}C$.

$$\delta^{13}C(CO_2) = \frac{\delta_1^{13}C(CO_2)\times[CO_2]_1+\delta_2^{13}C(CO_2)\times[CO_2]_2}{[CO_2]_1+[CO_2]_2} \qquad \text{(S4)}$$

where the subscript “1” denotes initial gases and “2” the second endmember denoting the product gases of relevance. The $\Delta_1^{17}O$ is the $\Delta^{17}O$ value of initial gases (O_3, O_2 from O_3 decomposition, or CO_2), $\Delta_2^{17}O$ is the value of newly formed molecules from water (see main text), $\delta_1^{13}C$ is the $\delta^{13}C$ value for initial CO_2, $\delta_2^{13}C$ is for new CO_2. In the current cases, $\Delta_1^{17}O(CO_2) = 0.2$ ‰, $\Delta_2^{17}O(CO_2) = \Delta_1^{17}O(O_3)$ or $3/4\times\Delta_1^{17}O(O_3)$, $\Delta_1^{17}O(O_2) = \Delta_1^{17}O(O_3)$, $\Delta_2^{17}O(O_2) = 0$ ‰, $[O_2]_2 = [CO_2]_2$, $\delta_1^{13}C(CO_2) = -33$ ‰, and $\delta_2^{13}C(CO_2) = -27$ ‰. The ensuing details for the isotope values taken follow. The expected values calculated using the equations are shown in Table S5. How the use of $\Delta^{17}O$ as a tracer facilitates the exploration of ozone reaction pathways is further explained in detail in the section on *O_3 oxidation systematics*.

Experimental setup

Gas-particle reaction experiments were conducted in a pre-cleaned and pre-evacuated glass system, following the design of Huang et al. [1] with modifications. The system was first checked and conditioned, to minimize ozone decay in the sample-free reaction vessel. The schematic diagram of the experimental setup is shown in Figure S1. Particle surface used for mediating heterogeneous oxygen isotope reaction between CO_2 and O_3 was provided by the particulate matters (PMs) collected on a filter paper in winter for one day in the main campus of Academia Sinica, Taipei, Taiwan, using a Tisch high-volume TSP aerosol sampler (for sampling details, see Guha et al. [2]).

In brief, the background air was pumped through a cleaned Whatman grade 41 cellulose filter paper (8″×10″); particles retained on the paper acted as reagents for the reactions explored in this study. A portion (1/8th of the filter, ~64 cm^2) of the filter paper was cut into pieces and placed in a glass reaction vessel (RB in Figure S1; volume ~60 ml). To explore homogeneity of PMs and sensitivity of surface water content on the processes studied, 4 reaction vessels (RB1-RB4) were prepared for the study. Prior to any of the experiments, each of the reaction vessels was exposed to the moist ambient air; this was done at the ambient room condition (7 cases) or air with an additional supply of water vapor (7 cases). Thereafter, the vessel was evacuated/degassed at ~5×10^{-6} torr for several hours until no further degassing could be noticed in an ion gauge (Duniway, I-075-NC, USA). This step removes a major part of the water vapor but not all and ensures the presence of a moderate amount of water on the particles.

The PM particles sampled in the region are highly complex compositionally [3]. They contain ~30% by mass of inorganic compounds and also ~30% of organics (sum of primary and secondary), with the remaining from sea salt, mineral dusts, and a large fraction of unidentified chemical components (~15%). In contrast, the previously explored PMs [4] or synthetic substances [5, 6] are less in organic contents and/or metal oxides. As a result, the O_3 reaction probability was not significant, unlike the high reactivity noted in this work and that found previously with liquid organics [7]. Following sections are an expanded discussion.

To explore the role of CO_2 in the reaction, aliquots of high purity CO_2 (>99.9999 %) were taken from a commercial cylinder (our lab standard tank AS-2) whose isotopic compositions ($\delta^{13}C$ = -32.7 ‰ VPDB, $\delta^{18}O$ = 36.3 ‰, and $\Delta^{17}O$ = 0.2 ‰) were determined *a priori* [8, 9]. O_3 was prepared using a tesla coil induced electric discharge in a flask (Buffer 2) filled with high purity oxygen (>99.999 %) where the produced ozone was collected immediately at liquid nitrogen temperature following Huang et al. [1]. After sufficient ozone (a few hundred μmole) was made, the remaining oxygen was pumped out. An aliquot of the ozone was taken in Buffer-1 from Buffer-2 and then transferred to a sample bottle containing 2 pellets of 1/16″ 5A molecular sieve; this breaks down the ozone and converts it to oxygen for isotope analysis. As expected, the ozone was enriched in heavy isotopes with $\delta^{18}O \approx$ 30-60 ‰ and $\Delta^{17}O \approx$ 7-17 ‰. About 60 μmole (~25 mbar in the line) of CO_2 and/or O_3, with known isotope ratios, were frozen and then released into the aforementioned reaction vessel (RB) containing the PMs, with and without CO_2 and allowed to

stay for about an hour to a few days (Tables S1-S3) in the dark (the line covered by black cloth). In a few cases only ozone or CO_2 was allowed to react with the airborne PMs (Table S1). After reaction, CO_2 was separated for triple oxygen isotopes and $\delta^{13}C$ determination.

CO_2 separation was initiated by transferring the mixture through U-traps at liquid nitrogen temperature where CO_2 and O_3 were retained. The product O_2 was frozen in a molecular sieve (2 pellets of 5A) containing bottle SB1 kept in liquid nitrogen. The CO_2+O_3 mixture was then transferred to a Ni containing vessel (Ni) at an elevated temperature of ~200 °C for 15 mins to decompose the O_3 to O_2, following Johnston et al. [10]. The CO_2 was retained by liquid N_2. After transferring the O_2 to SB1, the CO_2 was released and taken in SB2. In two cases (Expt. No. 38 and 39), the O_2 (from the remaining O_3) was taken in a separate sample bottle, allowing for separate isotope measurements of the product O_2 and unreacted O_3, if any. The isotopic analysis of O_2 was made on Thermo-Finnigan MAT 253, and precision for $\delta^{17}O$ and $\delta^{18}O$ was better than 0.1 ‰. The CO_2 $\delta^{17}O$ (and $\Delta^{17}O$) value was obtained using a platinum-catalyzed method [8, 11] with precision better than 0.1 ‰.

The sample size was estimated using the pressure gauge attached to the mass spectrometer and the relevant volumes. The sample bottles used for gas transfer vary between ~2 to 4 ml. The bellow size of the mass spectrometer is ~35 ml. In total, the sample size error was about 5 %. This error was taken into account when doing mass balance calculations for subsequent analysis and interpretation.

Hot nickel decomposition

We followed the method of Johnston et al. [10] to decompose O_3 to O_2 for O_3 oxygen isotopic analysis. Since the O_3 was admixed with CO_2 it was deemed important to examine whether the process would change the oxygen isotope composition of CO_2 and O_3. Table S1 summarizes the results. We see that the O_3 $\delta^{18}O$ does change which can be as much as ~5 ‰ but the $\Delta^{17}O$ changes are less than 0.3 ‰ and considered to be insignificant. In contrast, the CO_2 $\delta^{18}O$ changes are in general less than ~1 ‰ with $\Delta^{17}O$ changes of less than 0.5 ‰, except Expt. No. 6 where the $\Delta^{17}O$ change is ~0.7 ‰. We attribute these changes to improper handling during the recovery of samples

(the yield was not quantified), impurity in the reaction system, and possible isotope exchange between CO_2 and O_3 over hot nickel (see next paragraph). These are considered limitations of our method and introduce errors in the experimental results of PMs and O_3 reactions.

Regarding reactions of CO_2 with nickel at elevated temperature, it is possible that CO_2 could be decomposed to CO and O [12]. However, we did not observe presence of CO (at m/z 28); this suggests that either CO_2 was not decomposed or CO if any reacted subsequently. It is possible that the resulting CO and O, if present, could react with O_3, providing an explanation to the changes in the isotope compositions of CO_2 and O_3, in particular the positive changes in $\Delta^{17}O$ in CO_2.

For simple exposure (up to 60 hours) of CO_2 to PMs, we did not observe significant changes in its isotope composition, suggesting CO_2 is rather inert to PMs in ordinary conditions. However, we did notice that the final CO_2 $\Delta^{17}O$ could be depleted by as much as -0.3 ‰, compared to the initial value of 0.2 ‰. This could be due to isotope exchange between CO_2 and surface water [13] or improper handling of the CO_2.

Reactions of O_3 and PMs

Table S2 shows the reaction results of O_3 and PMs in presence of extra CO_2 (Expt. No. 12-37) and without it (Expt. No. 38-39). We did not have the amount quantified for all; the available ones are summarized in Table S3. The tables show that when ozone and CO_2 react in presence of aerosols, the isotope ratios of CO_2 and O_3 both change. These changes could be due to exchange of the initial CO_2 with ozone and/or formation of fresh CO_2 through ozone oxidation of organics which inherits ozone enrichment. Ozone oxidation indeed produces CO_2 as seen in two experiments done without any initial CO_2 (Expt. No. 38 and 39) where a large amount of fresh CO_2 (~18 μmole) formed in 48 hours. This demonstrates that ozone oxidizes organic matter lying on the particle surface to form CO_2. Notably, we see that CO_2 $\delta^{18}O$ values are depleted but their $\Delta^{17}O$ values are the same as that of O_3 (see also Table S4). In general, we notice that in this ozone oxidation experiment, CO_2 is the main product, and there is no CO. In two cases (Expt. No. 38 and 39), we monitored m/z 28 along with major beam at m/z 32 for the non-condensable gases (using liquid N_2) after the O_3-PMs reaction experiments. We find the m/z 28 signal is about 3% that of m/z 32,

implying ~1.5 μmol of m/z 28 gases which might be CO [14, 15]; it is also possible that the signal is due to modest air leakage or trapped air in the matrix during experiments.

The proposition of the formation of new CO_2 from O_3 oxidation is supported by the $\delta^{13}C$ and $\delta^{18}O$ values of CO_2. Figure S2 shows that the more is the CO_2 produced, the higher is the value of $\delta^{13}C$. Examining the isotope compositions of the CO_2 produced (Expt. No. 38 and 39), their $\delta^{13}C$ values are the same as the fossil fuel value (about -27 ‰) [9] from the region where PMs were collected for the experiments. The $\delta^{18}O$ values are around 35 ‰, ~20 ‰ lower than that of the initial O_3. This is explained by the preferential oxidation of lighter O_3 molecules (isotopocules) producing lighter CO_2 with an ^{18}O-fractionation factor around -20 ‰ (e.g., see ref. [1, 16]). The reactant O_3 becomes heavier in $\delta^{17,18}O$ during the course of the reaction but its $\Delta^{17}O$ value remains unchanged (Table S4).

Next, we examined whether the prior presence of CO_2 would interfere with the O_3 oxidation reactions. Experiments No. 12 to 37 (Table S3) were done to check if there is any effect of presence of some amount of initial CO_2 on the final CO_2 through possible exchange with O_3 proposed previously by Shaheen et al. [17] or other secondary processes. It is seen that the $\delta^{13}C$ and $\Delta^{17}O$ values both vary and they both correlate with the percentage of the excess CO_2 in the total (Figure S2 and main text Figure 1). This suggests that the isotope results can be explained by simple mixing between the freshly formed CO_2 and an unaltered initial CO_2. Therefore, the data does not support the mechanism of isotope exchange [13, 17]. For $\delta^{13}C$ the endmembers are -27 ‰ (from organics as expected) and -33 ‰ (from initial tank CO_2). For $\Delta^{17}O$ the endmembers are +0.2 ‰ (from initial tank CO_2) and $\Delta^{17}O$ value of the bulk O_3 or 3/4 of the bulk. With these endmembers, the observed CO_2 $\delta^{13}C$ and $\Delta^{17}O$ values are well represented (see main text Figure 1B, Figure S2B, and Table S5). Comparing cases with and without prior presence of CO_2, we notice one interesting phenomenon. Without prior presence of CO_2, the produced CO_2 has the same $\Delta^{17}O$ as the bulk O_3 $\Delta^{17}O$. However, with prior presence of CO_2, the produced CO_2 $\Delta^{17}O$ value tends to be 3/4 that of the bulk O_3 $\Delta^{17}O$ (see main text Figure 1), likely following the mechanism presented in the main text. The mechanism, however, provides a significant amount of CO that is not seen in the experiments. Nevertheless, CO_2 seems to play a role in the heterogeneous surface chemistry of ozone with organics, and further exploration of the role of CO_2 on the chemical kinetics is needed.

We can also infer the role of surface water in the reaction. We see from RB1 experiments (bottles with no extra water; Expt. No. 12-18), the reaction effect (O_3 oxidation) is marginal as reflected in the final CO_2 $\Delta^{17}O$ values. From Expt. No. 19 through 25, the reaction bottle was exposed to steam vapor, instead of typical room air. We see that the reaction effect is enhanced, as reflected in the final CO_2 $\Delta^{17}O$ values. This finding is also seen in RB2 (Expt. No.27-29). To further verify the role of water in the reaction, instead of exposing to steam water, we removed the moisture by heating the bottle at ~100 °C for ~30 mins until no degassing is seen. We observe that the O_3 oxidation was indeed minimal (Expt. No. 30) with the final CO_2 $\Delta^{17}O$ of 2.1 ‰. The reactivity could be partially restored when surface water is replaced; Expt. No. 31 demonstrates the case, where the experiment was done after exposing the reaction bottle to ambient air for 2 weeks after Expt. No. 30. The reason could be a loss of ozone ingression into the grains. Shiraiwa et al. [5] showed that the ozone uptake in aerosols exhibits a pronounced increase with relative humidity, which can be explained by a decrease of viscosity and increase of diffusivity of the host phase due to hygroscopic water uptake. Since the ozone uptake (reaction probability) is relatively minor at the surface [4-7, 18], the transfer of ozone from the surface to the near surface-bulk (where it can readily oxidize reactive components) determines the net uptake kinetics.

Another significant observation is that ozone decays slower in the presence of PMs than for the case where there is no PMs. Figure S3 shows the ozone decay in the reaction bottle without PMs; the exponential decay time is estimated to be about 10 hours. It shows that in PMs-free condition, after 48 hours, remaining ozone is less than ~1% of the initial amount. This is much smaller than the amount of O_3 remaining in the PMs-containing bottles (Expt. No. 38 and 39, where the remaining O_3 is ~20%). This contrast is counter-intuitive and suggests that in highly polluted regions, the O_3 lifetime can be greatly enhanced and this will facilitate the oxidation chemistry and associated adverse health impacts.

O_3 oxidation systematics

To further elucidate the ozone oxidation systematics some background information and current knowledge of ozone isotope transfer in oxidation processes are given below. It is known that ozone has an open isosceles triangular structure and the heavy isotopes are enriched in ozone relative to the oxygen gas used to form it by discharge (see Table S1). In addition, the enrichments in the two

heavy isotopes (^{17}O and ^{18}O) do not follow the usual mass dependent relation ($\delta^{17}O \approx 0.5\times\delta^{18}O$), i.e., the $\Delta^{17}O$ value is non-zero. It is also known that the anomalous enrichment occurs only in the terminal position and the $\Delta^{17}O$ in the central position is zero [19]. This means that the terminal $\Delta^{17}O$ value is 1.5 times the bulk $\Delta^{17}O$. It is important to note that in normal chemical reactions, the isotope $\Delta^{17}O$ value is constant which is a consequence of a mass-dependent process where $\delta^{17}O$ changes are half of $\delta^{18}O$ changes. Therefore, the $\Delta^{17}O$ value of the products can be used to trace the steps involved. However, for ozone there is an additional point to consider. It appears that conventionally, in the gas phase, ozone interacts mainly using the terminal position atoms. In such cases, the oxidation products would have a $\Delta^{17}O$ value higher than the bulk ozone [16, 20]. However, in heterogeneous reactions, ozone could behave in different ways, as seen in the experimental results presented in this work. The oxidation of inorganic and organic compounds (retained in the filter matrix) can occur via reaction with ozone, OH radicals, or a combination thereof, as discussed by von Gunten [21]. A similar mechanism was also proposed recently for explaining the experimental results of graphite ozone oxidation [14]. The reactions of ozone with inorganic compounds are typically fast and occur by an oxygen atom transfer reaction. In such cases, the atom would probably be from the terminal position (the product CO_2 $\Delta^{17}O$ values should then be 3/2 that of the initial bulk O_3) and the leftover O_2 would have a ^{17}O-excess equal to 3/4 of the bulk. This seems to be observed in $\Delta^{17}O$ of the product O_2 in Expt. No. 38 and 39. However, analysis of the other experiments shows that this is not the case. In addition, surprisingly, the CO_2 $\Delta^{17}O$ value is close to that of O_3.

The second mechanism is more complex and occurs by involving all three ozone atoms leading to isotopic scrambling, e.g., turning ordinary ozone to cyclic ozone [22]. In such case the product CO_2 would have a $\Delta^{17}O$ value nearly equal to the initial ozone value as seen in Expt. No. 38 and 39 but not for cases where CO_2 was present initially. We still need a mechanism to explain the observed depletion of $\Delta^{17}O$ in the product O_2. As mentioned in the main text, we think that this is due to addition of a certain fraction of O_2 from water.

Relation between O_2 and CO_2 isotope ratios as indicative of reaction pathways

Along with CO_2, there is significant production of O_2. The O_2 was collected along with O_3 remaining and we measured the O_2+O_3 together in most cases except two (Expts. No. 38 and 39)

where the remaining O_3 was separated from O_2. It appears that the total O_2 is composed of three components: (1) O_3 dissociation on the wall, (2) O_3 dissociation in the matrix, and (3) O_2 produced by ozone-mediated reactions in the matrix. The question arises as to the composition of this last O_2 because this cannot be isolated from the ozone derived O_2. However, we can get an idea of this by examining the balance of $\Delta^{17}O$ before and after the reaction. Figure S4 shows the changes of the total (sum of reactants or products measured) oxygen or O_2 $\Delta^{17}O$ values as a function of CO_2 $\Delta^{17}O$. Strangely, there is a reasonable anti-correlation between these two. We see from Figure S4A that the amount weighted changes in O_2 $\Delta^{17}O$ are nearly balanced by that in CO_2 (R^2=0.84), suggesting the in the final (O_2+O_3) there are two endmembers: ozone derived (O_2+O_3) having high $\Delta^{17}O$ (~12 ‰) without fractionation and another oxygen component with amount nearly equal to the new CO_2 but with low or zero $\Delta^{17}O$ (called New O_2). The Figure S4B supports this conclusion by showing that the more the depletion in O_2 $\Delta^{17}O$ (caused by addition of New O_2) the higher the enhancement in CO_2 $\Delta^{17}O$ (R^2=0.71) caused by addition of fresh CO_2. Indeed, two-component mass balance calculations show that if we assume the amount of New O2 to be equal to the amount of freshly produced CO_2 and if this fraction of O_2 possesses $\Delta^{17}O$ value of zero, the observed O_2 $\Delta^{17}O$ values can be explained (see main text and Figure 2B).

An obvious candidate to contribute zero $\Delta^{17}O$ in the New O_2 is water. A known pathway to produce O_2 from water is through OH reaction with O_3[7]. That, however, is insufficient to explain the hypothetical mechanism of the *total* O_2 production presented above, unless the O_2 is mainly from the OH pathway and that from O_3 decay is minimal (in that, O_2 would have a ^{17}O-excess equal to 3/4 of the bulk; see above for details). Instead, we hypothesize that the production is through the hypothetical R_1 and R_2 functional groups with surface water catalyzed by metal oxides present in the aerosol particles (see the reactions *R8-R10* discussed in the main text).

Which position

Which atom in an O_3 molecule takes part in ozone oxidation

We revisited the experimental data of S(IV) + O_3 by Savarino et al. [23], which tried to relate the resulting $\Delta^{17}O$ in sulfate to that in O_3. With a simple linear regression to their data, Savarino et al. [23] derived a coefficient of 0.24±0.02, assuming that all 3 oxygen atoms in O_3 contribute equally, in contrast to a numerical study by Liu et al. [24], who suggested terminal ozone participation. Both are used widely to assess the budget of sulfate in the atmosphere [25, 26]. Reanalysis of Savarino et al. [23] using Monte Carlo bootstrapping resampling approach (1000 times resampling), however, yields a slope of 0.38±0.87 (assuming bulk O_3 in the oxidation) or 0.26±0.58 (assuming terminal O_3 only). Both these values are indistinguishable from 0.25, expected for sulfate formation. Instead of performing linear regression analysis for $\Delta^{17}O(S(VI))$ vs. $\Delta^{17}O(O_3)$, if we try with the ratio of $\Delta^{17}O(S(VI))$ and $\Delta^{17}O(O_3)$, the results show that for bulk ozone the value is 0.35±0.06 and for the terminal ozone it is 0.23±0.04. Therefore, this analysis suggests that terminal ozone atom is responsible for the oxidation, favoring the scenario of Liu et al. [24]. However, within 2-σ error, the experiment does not yield a conclusive role of ozone in S(VI) oxidation. Further experiments are needed to elucidate the location of oxygen atoms in ozone molecule during oxidation, before a more accurate quantification of sulfate budget can be derived. This is an important issue because the S(IV)+O_3 process is generally believed to contribute ~25% of global sulfate production [26].

Chemistry-transport modeling

Here, we explore the environmental impact of the results of O_3 heterogeneous reactions using the Model for Ozone and Related chemical Tracers, version 4 (MOZART-4) [27]. The model is configured at T42 resolution and driven by the National Centers for Environmental Prediction analysis meteorology. We include the following two reactions in the model, following the same treatment as nitric acid heterogeneous processes:

$$O_3 + OC2 \rightarrow CO_2 + OC2$$

$$O_3 + SOA \rightarrow CO_2 + SOA$$

where OC2 and SOA are hydrophylic organic carbon and secondary organic aerosols, respectively. The reaction probability, γ_{O3}, is taken to be 0.001 (Figure 4 of the main text) or 0.0001 (Figure S5), in the range of ozonolysis of aqueous organics obtained previously [7]. The lower limit is chosen as 0.0001 below which the reaction becomes unimportant (see more below). Starting with the

initial concentrations (the meteorology data are for 2001-2002) the model reactions were allowed to proceed for two years and we take the second-year results for assessment and comparison. Overall, with this reaction probability and assuming the reaction is confined in the boundary 100 m, the process results in an annual carbon loss of ~7 TgC from PMs, which is significant compared to the organic aerosol production taken in the model [27].

Jacob (2000) also noted the importance of organic carbon aerosols on the loss of ozone: "One possibility in need of exploration is the role of organic carbon aerosols as sinks for O_3. Unsaturated organic compounds … high reactivity towards O_3 …." In this context, limited earlier studies suggested an upper limit of 10^{-4} [4, 18] for the reaction probability of ozone but also indicated that it could be as low as 10^{-8} rendering the process unimportant [18]. The controversy is as yet unresolved. The current study suggests that the reaction probability is significant and could lead to a major loss of ozone. Further analyses, in particular the PMs' particle distributions, are needed to quantify the value of the reaction probability, a quantity needed in chemistry-transport modeling, to assess the impact of the reaction in ozone budget and the oxidation state of organic aerosols.

References

(1) Huang, C. H.; Bhattacharya, S.; Hsieh, Z. M.; Chen, Y. J.; Yih, T. S.; Liang, M. C. Isotopic fractionation in photolysis of ozone in the Hartley and Chappuis Bands. *Earth and Space Science* **2019**, *6* (5), 752-773.
(2) Guha, T.; Lin, C.; Bhattacharya, S.; Mahajan, A.; Ou-Yang, C.-F.; Lan, Y.-P.; Hsu, S.; Liang, M.-C. Isotopic ratios of nitrate in aerosol samples from Mt. Lulin, a high-altitude station in Central Taiwan. *Atmospheric Environment* **2017**, *154*, 53-69.
(3) Chou, C. K.; Hsu, W. C.; Chang, S. Y.; Chen, W. N.; Chen, M. J.; Huang, W. R.; Huang, S. H.; Tsai, C. Y.; Chang, S. C.; Lee, C. T. Seasonality of the mass concentration and chemical composition of aerosols around an urbanized basin in East Asia. *Journal of Geophysical Research: Atmospheres* **2017**, *122* (3), 2026-2042.
(4) de Reus, M.; Dentener, F.; Thomas, A.; Borrmann, S.; Ström, J.; Lelieveld, J. Airborne observations of dust aerosol over the North Atlantic Ocean during ACE 2: Indications for heterogeneous ozone destruction. *Journal of Geophysical Research: Atmospheres* **2000**, *105* (D12), 15263-15275.
(5) Shiraiwa, M.; Ammann, M.; Koop, T.; Pöschl, U. Gas uptake and chemical aging of semisolid organic aerosol particles. *Proceedings of the National Academy of Sciences* **2011**, *108* (27), 11003-11008.

(6) Nicolas, M.; Ndour, M.; Ka, O.; D'Anna, B.; George, C. Photochemistry of atmospheric dust: ozone decomposition on illuminated titanium dioxide. *Environmental science & technology* **2009**, *43* (19), 7437-7442.
(7) de Gouw, J. A.; Lovejoy, E. R. Reactive uptake of ozone by liquid organic compounds. *Geophysical Research Letters* **1998**, *25* (6), 931-934.
(8) Mahata, S.; Bhattacharya, S. K.; Liang, M. C. An improved method of high-precision determination of Δ17O of CO2 by catalyzed exchange with O2 using hot platinum. *Rapid Communications in Mass Spectrometry* **2016**, *30* (1), 119-131.
(9) Laskar, A. H.; Mahata, S.; Liang, M.-C. Identification of anthropogenic CO_2 using triple oxygen and clumped isotopes. *Environmental science & technology* **2016**, *50* (21), 11806-11814.
(10) Johnston, J.; Röckmann, T.; Brenninkmeijer, C. CO_2+ O (^{1}D) isotopic exchange: Laboratory and modeling studies. *Journal of Geophysical Research: Atmospheres* **2000**, *105* (D12), 15213-15229.
(11) Mahata, S.; Bhattacharya, S. K.; Wang, C.-H.; Liang, M.-C. Oxygen isotope exchange between O_2 and CO_2 over hot platinum: An innovative technique for measuring $\Delta^{17}O$ in CO_2. *Analytical chemistry* **2013**, *85* (14), 6894-6901.
(12) Vogt, C.; Monai, M.; Sterk, E. B.; Palle, J.; Melcherts, A. E.; Zijlstra, B.; Groeneveld, E.; Berben, P. H.; Boereboom, J. M.; Hensen, E. J. Understanding carbon dioxide activation and carbon–carbon coupling over nickel. *Nature Communications* **2019**, *10* (1), 5330.
(13) Baltrusaitis, J.; Grassian, V. H. Surface reactions of carbon dioxide at the adsorbed water−iron oxide interface. *The Journal of Physical Chemistry B* **2005**, *109* (25), 12227-12230.
(14) Humeres, E.; de Castro, K. M.; Debacher, N. A.; Moreira, R. d. F. Reaction mechanism of the reduction of ozone on graphite. *Langmuir* **2020**, *36* (38), 11225-11236.
(15) Smith, D.; Welch, W.; Jassim, J.; Chughtai, A.; Stedman, D. Soot-ozone reaction kinetics: Spectroscopic and gravimetric studies. *Applied spectroscopy* **1988**, *42* (8), 1473-1482.
(16) Bhattacharya, S. K.; Savarino, J.; Michalski, G.; Liang, M.-C. A new feature in the internal heavy isotope distribution in ozone. *The Journal of Chemical Physics* **2014**, *141* (13), 134301.
(17) Shaheen, R.; Abramian, A.; Horn, J.; Dominguez, G.; Sullivan, R.; Thiemens, M. H. Detection of oxygen isotopic anomaly in terrestrial atmospheric carbonates and its implications to Mars. *Proceedings of the National Academy of Sciences* **2010**, *107* (47), 20213-20218.
(18) Stadtler, S.; Simpson, D.; Schröder, S.; Taraborrelli, D.; Bott, A.; Schultz, M. Ozone impacts of gas–aerosol uptake in global chemistry transport models. *Atmospheric chemistry and physics* **2018**, *18* (5), 3147-3171.
(19) Gao, Y. Q.; Marcus, R. Strange and unconventional isotope effects in ozone formation. *Science* **2001**, *293* (5528), 259-263. Michalski, G.; Bhattacharya, S. The role of symmetry in the mass independent isotope effect in ozone. *Proceedings of the National Academy of Sciences* **2009**, *106* (14), 5493-5496.
(20) Alexander, B.; Hastings, M.; Allman, D.; Dachs, J.; Thornton, J.; Kunasek, S. Quantifying atmospheric nitrate formation pathways based on a global model of the oxygen isotopic composition (Δ 17 O) of atmospheric nitrate. *Atmospheric Chemistry and Physics* **2009**, *9* (14), 5043-5056. Vicars, W. C.; Savarino, J. Quantitative constraints on the ^{17}O-excess ($\Delta^{17}O$) signature of surface ozone: Ambient measurements from 50 N to 50 S using the nitrite-coated filter technique. *Geochimica et Cosmochimica Acta* **2014**, *135*, 270-287.
(21) von Gunten, U. Ozonation of drinking water: part I. Oxidation kinetics and product formation. *Water Res* **2003**, *37* (7), 1443-1467. DOI: 10.1016/S0043-1354(02)00457-8.

(22) Burton, P. G.; Harvey, M. D. Theoretical evidence for metastable cyclic ozone. *Nature* **1977**, *266* (5605), 826-827. Flemmig, B.; Wolczanski, P. T.; Hoffmann, R. Transition metal complexes of cyclic and open ozone and thiozone. *Journal of the American Chemical Society* **2005**, *127* (4), 1278-1285.
(23) Savarino, J.; Lee, C. C.; Thiemens, M. H. Laboratory oxygen isotopic study of sulfur (IV) oxidation: Origin of the mass-independent oxygen isotopic anomaly in atmospheric sulfates and sulfate mineral deposits on Earth. *Journal of Geophysical Research: Atmospheres* **2000**, *105* (D23), 29079-29088.
(24) Liu, Q.; Schurter, L. M.; Muller, C. E.; Aloisio, S.; Francisco, J. S.; Margerum, D. W. Kinetics and mechanisms of aqueous ozone reactions with bromide, sulfite, hydrogen sulfite, iodide, and nitrite ions. *Inorganic Chemistry* **2001**, *40* (17), 4436-4442.
(25) Shao, C.; Yu, B.; Liu, Y. Androgen receptor splicing variant 7: Beyond being a constitutively active variant. *Life sciences* **2019**, *234*, 116768. Itahashi, S.; Hattori, S.; Ito, A.; Sadanaga, Y.; Yoshida, N.; Matsuki, A. Role of Dust and Iron Solubility in Sulfate Formation during the Long-Range Transport in East Asia Evidenced by 17O-Excess Signatures. *Environmental science & technology* **2022**, *56* (19), 13634-13643.
(26) Alexander, B.; Park, R. J.; Jacob, D. J.; Gong, S. Transition metal-catalyzed oxidation of atmospheric sulfur: Global implications for the sulfur budget. *Journal of Geophysical Research: Atmospheres* **2009**, *114* (D2).
(27) Emmons, L. K.; Walters, S.; Hess, P. G.; Lamarque, J.-F.; Pfister, G. G.; Fillmore, D.; Granier, C.; Guenther, A.; Kinnison, D.; Laepple, T. Description and evaluation of the Model for Ozone and Related chemical Tracers, version 4 (MOZART-4). *Geoscientific Model Development* **2010**, *3* (1), 43-67.

Table S1: Control experiments of CO_2 and O_3 with hot nickel or particulate matters (PMs) done with reaction vessel RB1

Expt. #	Reaction time (hr)	Initial O_3		Final O_3+O_2		Final CO_2		
		$\delta^{18}O$	$\Delta^{17}O$	$\delta^{18}O$	$\Delta^{17}O$	$\delta^{13}C$	$\delta^{18}O$	$\Delta^{17}O$
**<u>CO_2 or O_3 with hot nickel</u>**								
1	0	…	…	…	…	-32.9	37.0	0.2
2	0	55.7	13.8	57.8	13.8	…	…	…
3	0	56.8	13.9	57.6	14.0	…	…	…
4	0	48.2	10.6	50.3	10.6	-33.1	37.1	0.7
5	0	51.7	12.3	50.6	12.0	-32.9	37.4	0.7
6	0	54.1	13.1	58.8	13.2	-33.0	37.6	0.9
7	0	54.6	13.0	55.8	13.1	-32.9	37.4	0.7
**<u>CO_2 with PMs</u>**								
8	24	…	…	…	…	-32.5	36.5	-0.1
9	24	…	…	…	…	-32.9	36.5	0.1
10	60	…	…	…	…	-32.9	36.9	0.2
11	60	…	…	…	…	-32.9	37.0	0.3

Table S2: Isotopic composition of CO_2, O_3, and particulate matter (PMs) before and after reactions executed in 4 different reaction vessels containing the same quantity of PMs (RB1 to RB4). Prior to each of Expt. No. 12-18, RB1 was exposed to the ambient room air. For Expt. No. 19-25, RB1 was exposed to steam (indicated by superscript "w"). RB1 conditions were repeated in RB2 to RB4 to verify the results derived. To examine the role of water in the reaction, Expt. No. 30 (with superscript "d") was done by heating RB2 at ~100℃ for ~30 min to drive away water. Expt. No. 31 was done after exposing RB2 to the ambient room air for 2 weeks.

Expt. #	Vessel# /Humid	Reaction time (hr)	Initial O_3		Final O_3+O_2		Final CO_2		
			$\delta^{18}O$	$\Delta^{17}O$	$\delta^{18}O$	$\Delta^{17}O$	$\delta^{13}C$	$\delta^{18}O$	$\Delta^{17}O$
**Mixture of CO_2 and O_3 with PMs**									
12	RB1	24	N/A	N/A	N/A	N/A	-31.5	36.7	3.7
13	RB1	24	N/A	N/A	N/A	N/A	-31.8	36.2	2.8
14	RB1	1	50.3	15.0	50.4	14.5	-32.7	37.0	1.2
15	RB1	3	50.1	14.5	47.6	13.6	-32.5	37.0	1.6
16	RB1	14	38.8	13.7	44.1	11.9	-32.4	36.3	1.8
17	RB1	24	35.0	10.8	33.7	10.0	-32.5	36.5	1.0
18	RB1	24	39.8	12.2	38.9	11.5	-32.6	36.5	1.0
19[w]	RB1[w]	24	38.7	11.2	34.2	9.6	-32.3	36.7	1.7
20[w]	RB1[w]	24	32.7	9.6	33.8	8.4	-32.4	36.2	1.3
21[w]	RB1[w]	24	34.2	10.4	35.4	9.3	-32.4	35.6	1.5
22[w]	RB1[w]	60	49.0	11.0	49.8	9.0	-32.5	37.0	2.6
23[w]	RB1[w]	60	52.1	12.5	55.0	10.4	-32.1	37.5	2.5
24[w]	RB1[w]	60	53.8	13.1	58.8	11.8	-32.3	37.0	2.0
25[w]	RB1[w]	60	57.1	13.8	59.6	12.7	-32.2	37.5	2.3
26	RB2	24	N/A	N/A	N/A	N/A	-32.2	36.8	2.7
27	RB2	1	55.7	17.2	55.2	16.2	-32.5	36.8	1.3
28	RB2	3	52.5	14.3	44.9	13.0	-32.4	36.7	1.5
29	RB2	24	40.3	12.6	39.3	11.5	-32.5	36.3	1.5
30[d]	RB2[d]	144	44.2	9.2	44.1	8.0	-32.3	37.3	2.1
31[d*]	RB2[d]	24	48.4	10.9	47.6	10.4	-32.9	36.7	1.6
32	RB3	24	41.2	7.2	40.0	4.9	-32.6	36.4	2.3
33	RB3	24	51.4	12.0	47.1	7.3	-31.9	37.1	3.4
34	RB3	24	48.5	11.1	52.7	8.0	-32.3	35.2	3.6
35	RB4	24	N/A	N/A	N/A	N/A	-32.6	36.3	2.2
36	RB4	24	43.7	9.2	48.3	6.2	-31.6	35.0	3.2
37	RB4	24	47.0	10.3	52.4	7.0	-32.1	34.2	3.6
**O_3 with PMs**									
38	RB3	48	52.7	12.8	56.6	9.5	-27.3	35.3	12.8
39	RB4	48	56.7	13.9	61.5	9.9	-26.7	34.8	14.8

Table S3: Results of the experiments where the amounts of reactants were measured. ΔAmnt denotes the excess of CO_2 over the initial amount

Expt. #	Bottle# /Humid	Reaction time (hr)	Initial O_3			Final O_2, O_3			Initial CO_2	Final CO_2				
			Amnt.	$\delta^{18}O$	$\Delta^{17}O$	Amnt.	$\delta^{18}O$	$\Delta^{17}O$	Amnt.	Amnt.	ΔAmnt	$\delta^{13}C$	$\delta^{18}O$	$\Delta^{17}O$
17	RB1	24	95	35.0	10.8	84	33.7	10	65	65	0	-32.5	36.5	1.0
18	RB1	24	92	39.8	12.2	85	38.9	11.5	65	68	3	-32.6	36.5	1.0
22[w]	RB1w	60	91	49.0	11.0	81	49.8	9.0	63	69	6	-32.5	37	2.6
23[w]	RB1w	60	94	52.1	12.5	80	55.0	10.4	67	78	11	-32.1	37.5	2.5
24[w]	RB1w	60	94	53.8	13.1	82	58.8	11.8	66	74	8	-32.3	37	2.0
25[w]	RB1w	60	94	57.1	13.8	85	59.6	12.7	65	75	10	-32.2	37.5	2.3
29	RB2	24	90	40.3	12.6	77	39.3	11.5	60	65	5	-32.5	36.3	1.5
30[d]	RB2d	144	94	44.2	9.2	83	44.1	8.0	63	75	12	-32.3	37.3	2.1
31[d*]	RB2d	24	91	48.4	10.9	85	47.6	10.4	63	71	8	-32.9	36.7	1.6
32	RB3	24	119	41.2	7.2	90	40.0	4.9	67	78	11	-32.6	36.4	2.3
34	RB3	24	90	48.5	11.1	71	52.7	8.0	62	81	18	-32.3	35.2	3.6
36	RB4	24	99	43.7	9.2	81	48.3	6.2	63	83	20	-31.6	35	3.2
37	RB4	24	102	47.0	10.3	77	52.4	7.0	62	86	24	-32.1	34.2	3.6
38	RB3	48	87	52.7	12.8	67	56.6	9.5	0	18	18	-27.3	35.3	12.8
39	RB4	48	83	56.7	13.9	64	61.6	9.9	0	19	19	-26.7	34.8	14.8

Table S4 Results of the experiments where O_3 and ***<u>O</u>***$_2$ were separated

Expt. #	Bottle# /Humid	Reaction time (hr)	Initial O_3			Final ***O_2***, O_3			Initial CO_2	Final CO_2				
			Amnt.	$\delta^{18}O$	$\Delta^{17}O$	Amnt.	$\delta^{18}O$	$\Delta^{17}O$	Amnt.	Amnt.	ΔAmnt	$\delta^{13}C$	$\delta^{18}O$	$\Delta^{17}O$
38	RB3	48	87	52.7	12.8	***52***<br>15	***48.2***<br>85.9	***8.5***<br>12.9	0	18	18	-27.3	35.3	12.8
39	RB4	48	83	56.7	13.9	***51***<br>13	***53.3***<br>94.1	***8.8***<br>14.2	0	19	19	-26.7	34.8	14.8

Table S5: Measured vs. calculated (see equations S2-S4) isotope values (in ‰ VPDB or VSMOW). The calculated values for CO_2 assume that the newly produced CO_2 from the ozone reaction has the same $\Delta^{17}O$ value as that of O_3

Expt. #	Final CO_2				Final $O_2 + O_3$	
	$\delta^{13}C$		$\Delta^{17}O$		$\Delta^{17}O$	
	measured	calculated	measured	calculated	measured	calculated
17	-32.5	-33.0	1.0	0.2	10.0	10.8
18	-32.6	-32.7	1.0	0.7	11.5	11.8
22	-32.5	-32.5	2.6	1.1	9.0	10.2
23	-32.1	-32.2	2.5	1.9	10.4	10.8
24	-32.3	-32.4	2.0	1.6	11.8	11.8
25	-32.2	-32.2	2.3	2.0	12.7	12.2
29	-32.5	-32.5	1.5	1.2	11.5	11.8
30	-32.3	-32.0	2.1	1.6	8.0	7.9
31	-32.9	-32.3	1.6	1.4	10.4	9.9
32	-32.6	-32.2	2.3	1.2	4.9	6.3
34	-32.3	-31.3	3.6	2.6	8.0	8.3
36	-31.6	-31.6	3.2	2.4	6.2	6.9
37	-32.1	-31.3	3.6	3.0	7.0	7.1
38	-27.4	-27.0	12.8	12.8	11.2	9.4
39	-26.7	-27.0	14.8	13.9	10.4	9.8

Figure S1: Schematic diagram of the reaction system

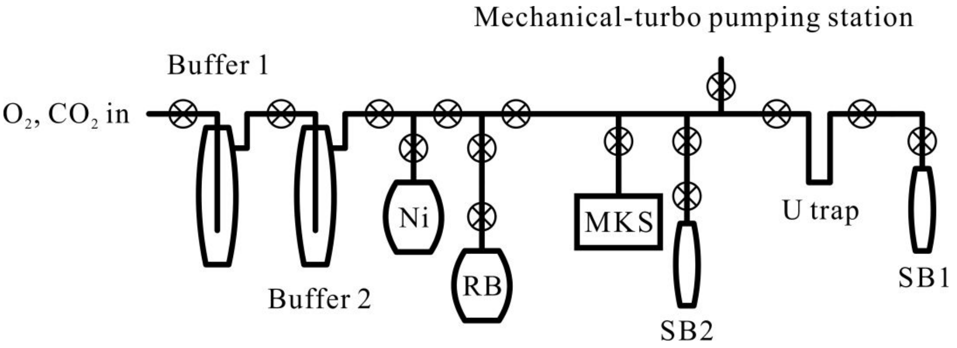

Figure S2: (A) The correlation between the CO_2 $\delta^{13}C$ and the ratio of the excess CO_2 versus total CO_2. (B) The measured vs. calculated $\delta^{13}C$ using a two end-member mixing model (described in the text). When the ratio is unity the $\delta^{13}C$ has the value -27 ‰, equal to that of the organic carbon in the matrix, as expected. When the ratio is smaller the added CO_2 changes the $\delta^{13}C$ value of the final CO_2 proportionately to near the tank value -32.5 ‰.

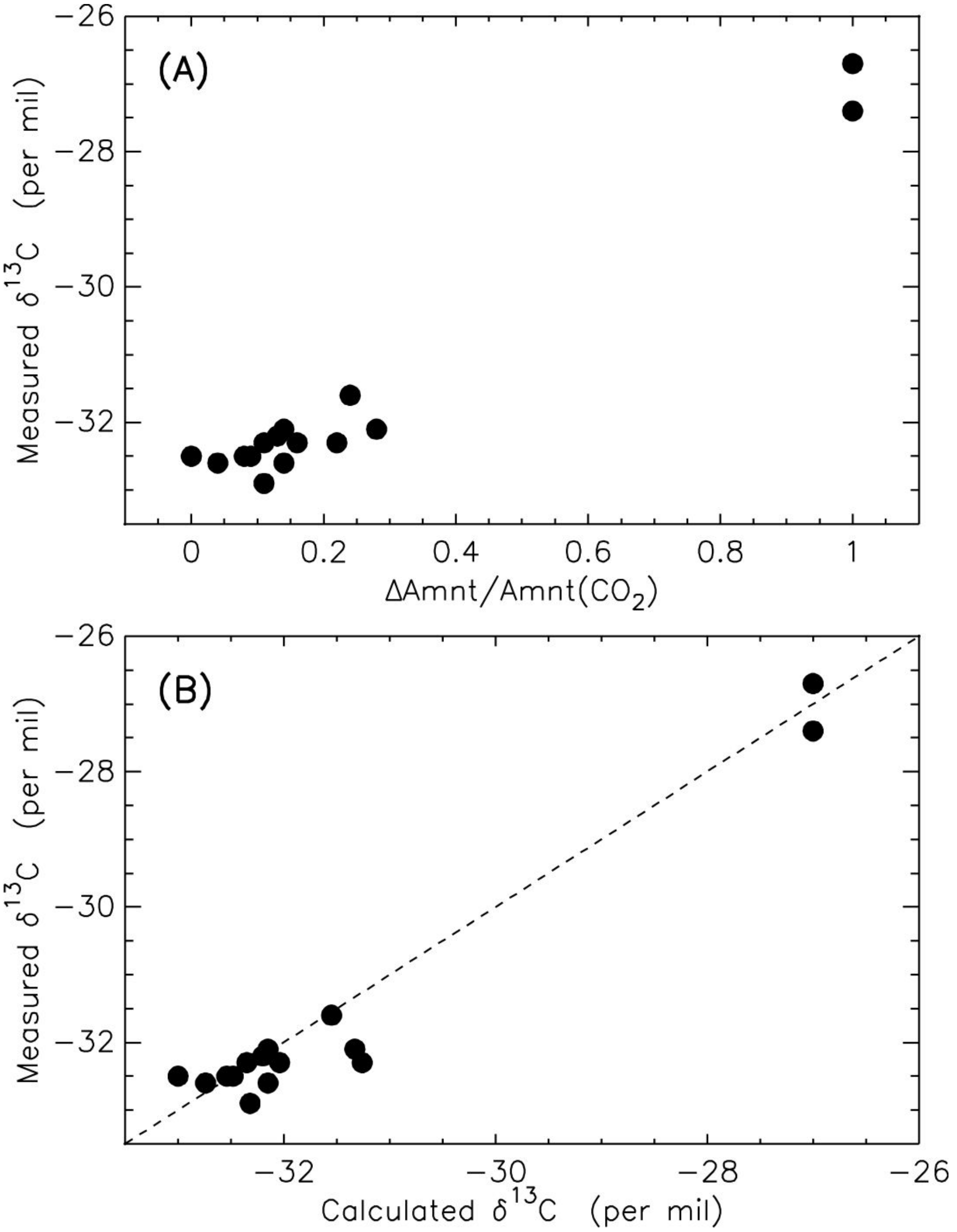

Figure S3: The decay of ozone in a PM_s-free sample bottle. The best fitted exponential decay time is 9±1 hours. The remaining O_3 fraction in a PMs-containing vessel after 48 hours of O_3 + PMs reaction is shown by the solid red circles (data from Expt. No. 38 and 39).

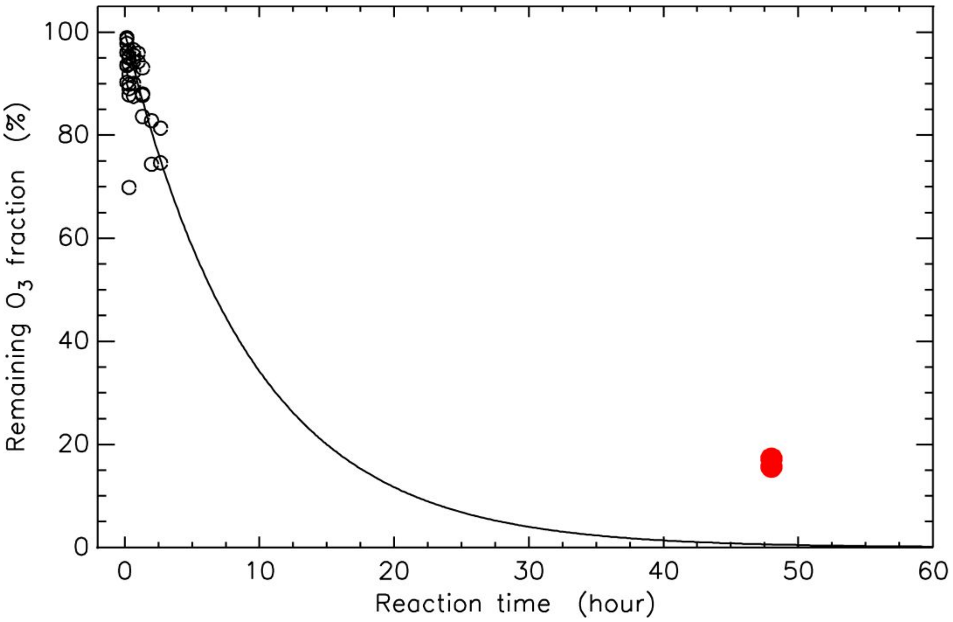

Figure S4: (A) Changes of concentration (total O_2+O_3) weighted O_2 $\Delta^{17}O$ (μmole ‰) versus concentration weighted CO_2 $\Delta^{17}O$ changes (μmole ‰) (data from Table S3). The R^2 value of the least-squared linear regression with zero intercept is 0.84. (B) Changes in O_2 $\Delta^{17}O$ versus CO_2 $\Delta^{17}O$ values. The R^2 value of the least-squared linear regression with zero intercept is 0.71.

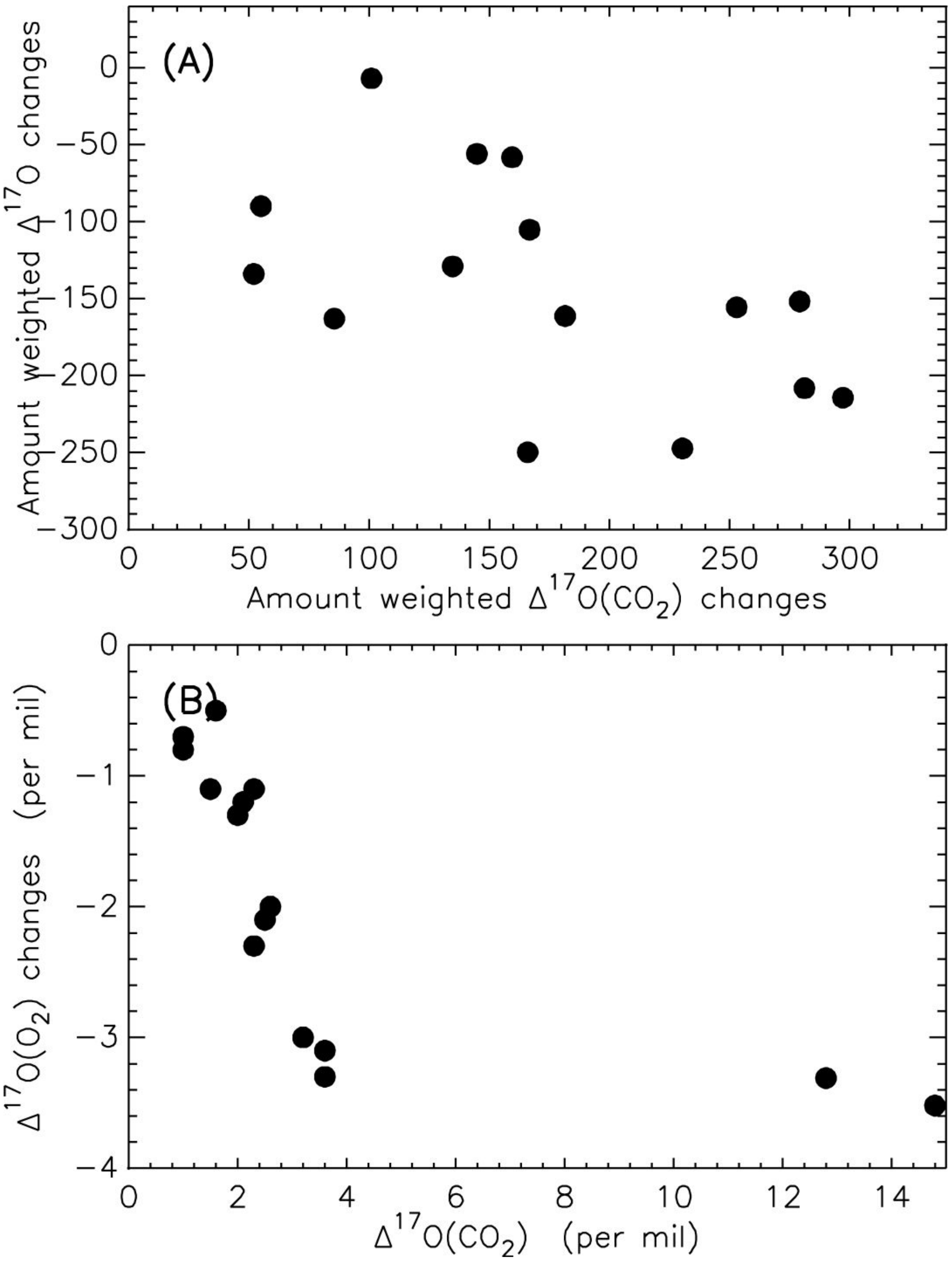

Figure S5: MOZART-4 simulation results with the inclusion of O_3 + PMs with the reaction probability γ_{O3} of 0.0001. **(Top)** The annually averaged heterogeneous reaction rate (μgC/m^3/day) that oxidizes PMs to CO_2. **(Middle)** The annually averaged surface O_3 concentration (ppb). **(Bottom)** The annually averaged surface O_3 concentration (ppb) from the standard model. (The figures are made using GEOV geophysical visualization tool. Copyright: University Corporation for Atmospheric Research and Max Planck Institute for Meteorology, Hamburg.)

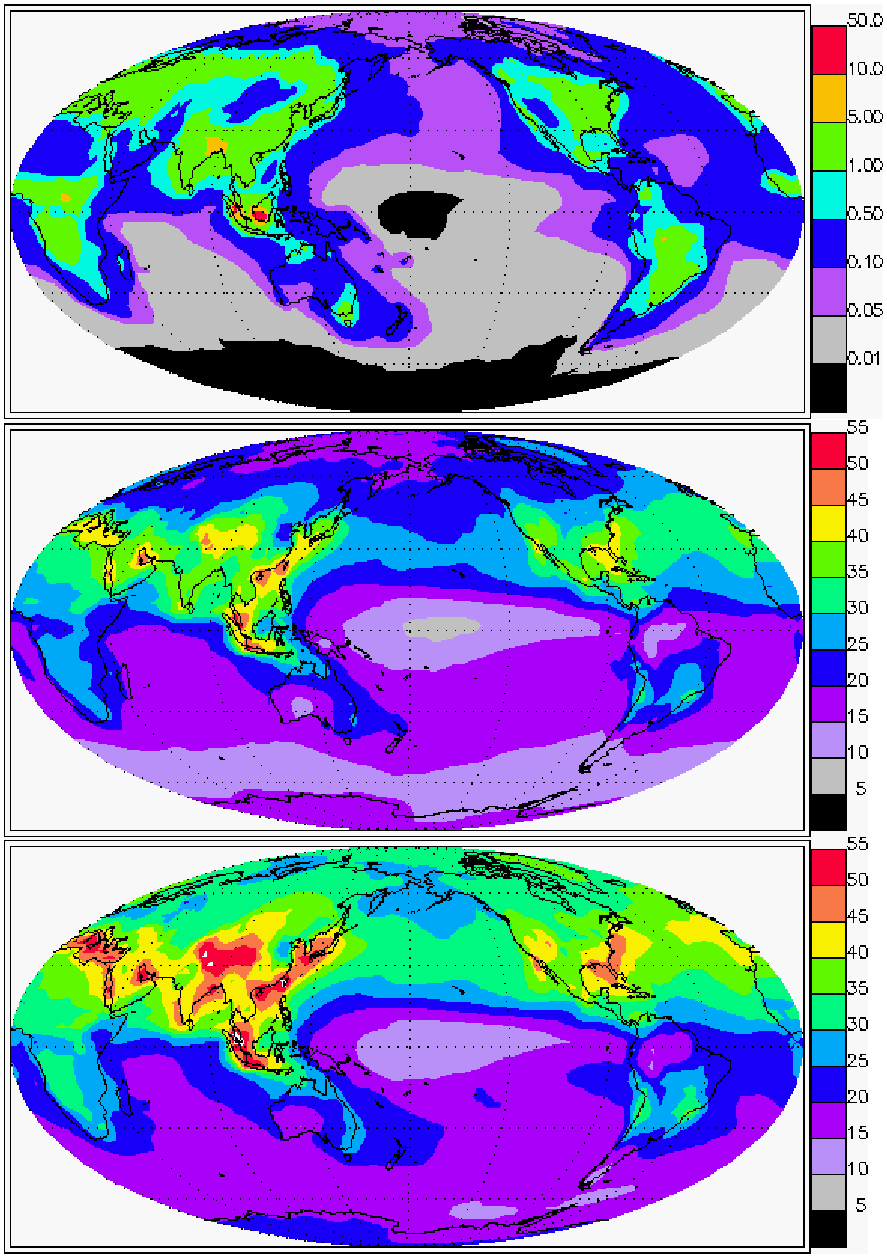

Supplement: Supplementary file 1 — ao4c06957_si_001.pdf [file ao4c06957_si_001.pdf]
